# Spontaneous Autoimmunity in 129 and C57BL/6 Mice—Implications for Autoimmunity Described in Gene-Targeted Mice

**DOI:** 10.1371/journal.pbio.0020243

**Published:** 2004-08-17

**Authors:** Anne E Bygrave, Kirsten L Rose, Josefina Cortes-Hernandez, Joanna Warren, Robert J Rigby, H. Terence Cook, Mark J Walport, Timothy J Vyse, Marina Botto

**Affiliations:** **1**Rheumatology Section, Eric Bywaters CentreImperial College, London, United Kingdom; **2**Department of Histopathology, Faculty of MedicineImperial College, LondonUnited Kingdom

## Abstract

Systemic lupus erythematosus (SLE) is a multisystem autoimmune disorder in which complex genetic factors play an important role. Several strains of gene-targeted mice have been reported to develop SLE, implicating the null genes in the causation of disease. However, hybrid strains between 129 and C57BL/6 mice, widely used in the generation of gene-targeted mice, develop spontaneous autoimmunity. Furthermore, the genetic background markedly influences the autoimmune phenotype of SLE in gene-targeted mice. This suggests an important role in the expression of autoimmunity of as-yet-uncharacterised background genes originating from these parental mouse strains. Using genome-wide linkage analysis, we identified several susceptibility loci, derived from 129 and C57BL/6 mice, mapped in the lupus-prone hybrid (129 × C57BL/6) model. By creating a C57BL/6 congenic strain carrying a 129-derived Chromosome 1 segment, we found that this 129 interval was sufficient to mediate the loss of tolerance to nuclear antigens, which had previously been attributed to a disrupted gene. These results demonstrate important epistatic modifiers of autoimmunity in 129 and C57BL/6 mouse strains, widely used in gene targeting. These background gene influences may account for some, or even all, of the autoimmune traits described in some gene-targeted models of SLE.

## Abstract 2

Several strains of gene-targeted mice develop systemic lupus erythematosus (SLE). Analysis of these strains demonstrates that the genetic background profoundly influences the development of autoimmunity

## Introduction

Systemic lupus erythematosus (SLE) is a chronic autoimmune disease characterised by the production of autoantibodies (auto-Abs) against a wide spectrum of self-antigens, mainly from subcellular compartments, especially the cell nucleus. Genetic predisposition is an important contributor to susceptibility to SLE in both humans and animals (Vyse and Todd 1996; Harley et al. 1998; Theofilopoulos and Kono 1999; Wakeland et al. 2001). Genes in multiple pathways participate in mediating disease pathogenesis, and epistatic interactions amongst these genes influence the expression of disease. In this context, both genetic linkage studies in spontaneous lupus-prone models and synthetic murine models of autoimmunity generated by targeted disruption of specific genes modulating the immune system have widely been used to investigate the complexity of SLE.

The best-studied strains of mice that spontaneously develop a lupus-like pathology are the New Zealand Black/New Zealand White hybrid strain (NZB/WF1); the MRL/Mp *lpr/lpr* strain, which carries the *lpr* mutation of the FAS receptor gene; and the BXSB strain, which carries the Y chromosome autoimmune accelerator (*Yaa*) gene (Theofilopoulos and Dixon 1985). Extensive genetic mapping studies in all three strains have identified multiple intervals associated with disease susceptibility. Interestingly, the majority of the intervals detected are strain-specific, confirming the genetic complexity of the disease and indicating the presence of extensive heterogeneity in the genes contributing to the pathogenesis of the disease. However, some susceptibility loci have been mapped to similar chromosome locations in different strains, suggesting that at least some susceptibility may be shared amongst lupus-prone strains. Amongst these shared susceptibility loci, the most striking are loci on distal Chromosome 1, for which important contributing genes have been found in New Zealand and BXSB models (Theofilopoulos and Kono 1999; Wakeland et al. 2001).

Although considerable efforts have been made to identify the genes responsible for the development of the disease, with the exception of the *lpr* mutation, none of the genetic contributions to disease in the three well-documented murine SLE strains have yet been fully resolved at the molecular or protein level. Thus, targeted genetic disruption of candidate genes encoding proteins of the immune system has been extensively used to examine their role in immune regulation. However, the most surprising result of this powerful approach has been the high frequency with which such mutations have been associated with an autoimmune phenotype. In this regard, it is of note that hybrid strains between 129 and C57BL/6 mice, widely used in the generation of gene-targeted mice, are spontaneously predisposed to development of humoral autoimmunity with low levels of glomerulonephritis (Obata et al. 1979; Botto et al. 1998; Bickerstaff et al. 1999; Santiago-Raber et al. 2001). Furthermore, the genetic background markedly influences the autoimmune phenotype in gene-targeted mice (Bolland and Ravetch 2000; Santiago-Raber et al. 2001; Mitchell et al. 2002). These observations led to the hypothesis that the autoimmune phenotype described in some gene-targeted mice might be due primarily to combinations of as-yet-uncharacterised background genes, originating from 129 and C57BL/6 mice strains, interacting or not with the mutated allele. To test this, we conducted a genome-wide scan analysis of two large cohorts of (129 × C57BL/6)F2 mice, one of which carried a mutation in the serum amyloid P component gene (*Apcs*). The *Apcs*-deficient mice (*Apcs* ^−/−^) were chosen as an example of a gene-targeted strain previously reported to develop a lupus-like disease on the hybrid genetic background (129 × C57BL/6); autoimmunity in *Apcs* ^−/−^ mice persists even after backcrossing the mutated gene onto C57BL/6 (Bickerstaff et al. 1999). We chose this targeted gene in particular to study since the *Apcs* gene is located on Chromosome 1, approximately 94 cM from the centromere, within a region where several lupus-susceptibility loci, designated *Sle1* (Morel et al. 2001), *Nba2* (Drake et al. 1995; Vyse et al. 1997), and *Bxs3* (Hogarth et al. 1998; Haywood et al. 2000), have been mapped in NZW, NZB, and BXSB lupus-prone strains, respectively. This region contains several genes, including those encoding FcγRII, the complement receptor CR1/2 (CD35/CD21), and the decay-accelerating factor CD55 (Prodeus et al. 1998; Bolland and Ravetch 2000; Miwa et al. 2002; Wu et al. 2002), which have each been implicated in the causation of SLE when inactivated by gene-targeting in 129 embryonic stem cells.

Here we show that there are multiple genetic loci, derived from both 129 and C57BL/6 mice, contributing to autoimmunity. Furthermore, a 129-derived interval on distal Chromosome 1, when transferred onto the C57BL/6 genome, a combination commonly created by backcrossing onto C57BL/6 a gene that has been inactivated in 129 embryonic stem cells, was sufficient to cause humoral autoimmunity in its own right, irrespective of the presence of the mutated *Apcs* gene. These results demonstrate important epistatic interactions between genes from 129 and C57BL/6 genomes on the development of autoimmunity and illustrate the important effects of background genes in the analysis and interpretation of autoimmune phenotypes associated with targeted genetic disruptions.

## Results

### Disease Traits in (129 × C57BL/6)F2 and (129 × C57BL/6)F2.*Apcs* ^−/−^ Mice

To investigate the genetic basis of the lupus-like disease observed in the (129 × C57BL/6) hybrid mice, we generated two large cohorts of (129 × C57BL/6)F2 animals, one carrying a mutation in the *Apcs* gene, and monitored them for 1 y under identical environmental conditions. Since female mice in the original reports showed a higher penetrance of disease, the present study was conducted only on female mice. The results of the phenotypic analysis at 1 y of age are summarised in Tables 1 and 2. As previously reported (Botto et al. 1998), the wild-type (129 × C57BL/6)F2 mice developed lupus traits with elevated levels of auto-Abs, starting from 6 mo of age (data not shown), and histological evidence of proliferative glomerulonephritis. In agreement with our previous observations (Bickerstaff et al. 1999), the titres of anti-nuclear Abs (ANAs) and anti-chromatin Ab were considerably greater in the (129 × C57BL/6)F2.*Apcs* ^−/−^ mice compared with the strain-matched controls. However, in contrast to our original findings, the levels of the other two disease serological markers analysed (anti-single-stranded DNA [ssDNA] and anti-double-stranded DNA [dsDNA] Abs) and the severity of the renal pathology were not different between the two experimental groups. In view of the possibility of an association between the fixed 129-derived segment flanking the mutated *Apcs* gene and the autoimmune traits observed, the genome-wide linkage analysis of the two experimental cohorts was carried out separately.

**Table 1:**
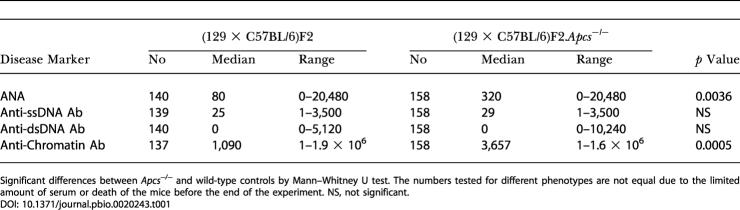
Spontaneous Auto-Abs in *Apcs* ^−/−^ and Wild-Type (129 × C57BL/6)F2 Female Mice

Significant differences between *Apcs* ^−/−^ and wild-type controls by Mann–Whitney U test. The numbers tested for different phenotypes are not equal due to the limited amount of serum or death of the mice before the end of the experiment. NS, not significant

**Table 2:**
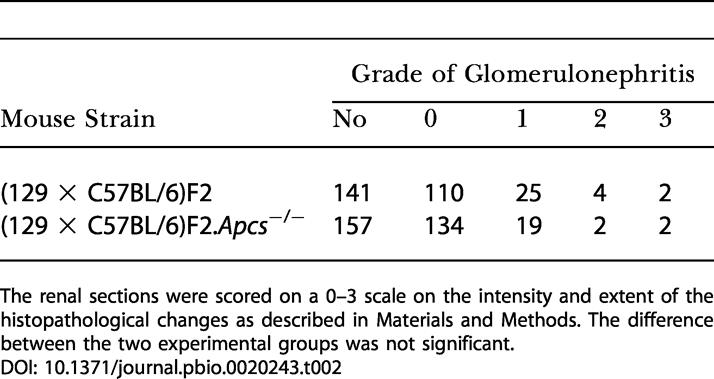
Histological Assessment of Kidney Sections in *Apcs* ^−/−^ and Wild-Type (129 × C57BL/6)F2 Female Mice

The renal sections were scored on a 0–3 scale on the intensity and extent of the histopathological changes as described in Materials and Methods. The difference between the two experimental groups was not significant

### Mapping of Loci Predisposing to Lupus in the Hybrid Strain (129 × C57BL/6)

**Figure 1:**
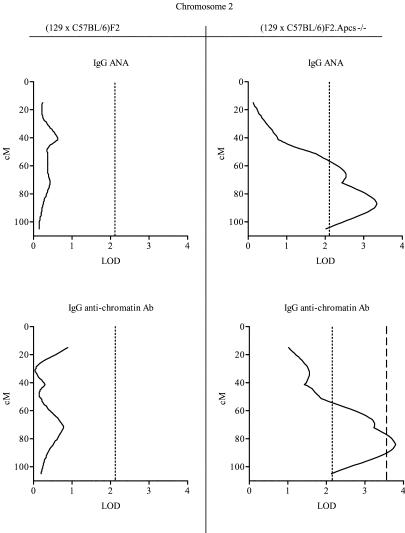
Linkage of Chromosome 2 with ANA and Anti-Chromatin Abs in (129 × C57BL/6)F2*.Apcs^−/−^* Mice These associations were not detected in (129 × C57BL/6)F2 animals. Centimorgan positions were deduced by interval mapping, anchoring marker locations to data from http://www.informatics.jax.org. Dotted lines and the dashed line indicate the threshold over which linkages were considered suggestive or significant, respectively, as defined in Materials and Methods.

**Figure 2:**
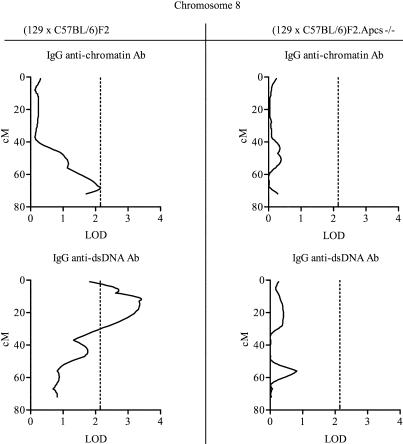
Linkage of Chromosome 8 with Anti-Chromatin and Anti-dsDNA Abs in (129 × C57BL/6)F2 Mice These linkages were not detected in (129 × C57BL/6)F2*.Apcs^−/−^* animals. Centimorgan positions were deduced by interval mapping, anchoring marker locations to data from http://www.informatics.jax.org. Dotted lines indicate the threshold over which linkage was considered suggestive, as defined in Materials and Methods.

**Figure 3:**
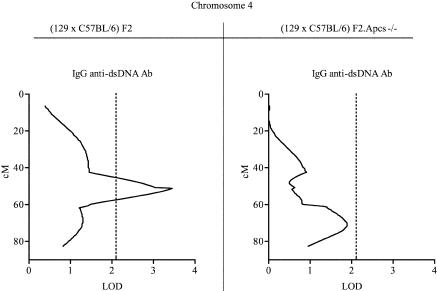
Linkage of Chromosome 4 with Anti-dsDNA Abs The estimated peak in (129 × C57BL/6)F2 mice was at position 51.3 cM, whilst in the (129 × C57BL/6)F2*.Apcs^−/−^*animals it was was at position 71 cM, indicating that most likely these were two independent loci. Centimorgan positions were deduced by interval mapping, anchoring marker locations to data from http://www.informatics.jax.org. Dotted lines the indicate threshold over which linkage was considered suggestive, as defined in Materials and Methods**.**

Mice were genotyped with 143 microsatellite markers distributed throughout the autosomes such that 98% of the genes were within 20 cM of an informative marker. A summary of the genome-wide linkage analysis for each of the disease traits measured is shown in Table 3. The areas of linkage were defined according to the parental origin, 129 or C57BL/6. Only linkages identified in both experimental groups are reported in Table 3, with the exception of the Chromosome 1 distal segment, where the linkage analysis could not be applied to the (129 × C57BL/6)F2.*Apcs* ^−/−^ mice as this region was of fixed 129 origin. Chromosomes where linkages were present only in one of the two cohorts are shown in Figures 1–3.

**Table 3:**
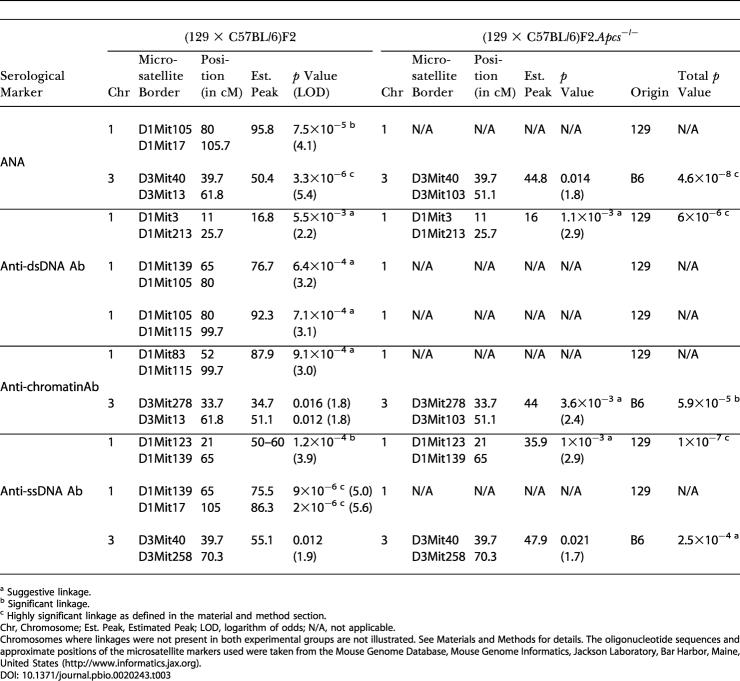
Summary of Genome-Wide Linkage Analysis in *Apcs* ^−/−^ and Wild-Type (129 × C57BL/6)F2 Female Mice

^a^ Suggestive linkage

^b^ Significant linkage

^c^ Highly significant linkage as defined in the material and method section

Chr, Chromosome; Est. Peak, Estimated Peak; LOD, logarithm of odds; N/A, not applicable

Chromosomes where linkages were not present in both experimental groups are not illustrated. See Materials and Methods for details. The oligonucleotide sequences and approximate positions of the microsatellite markers used were taken from the Mouse Genome Database, Mouse Genome Informatics, Jackson Laboratory, Bar Harbor, Maine, United States (http://www.informatics.jax.org)

**Figure 4:**
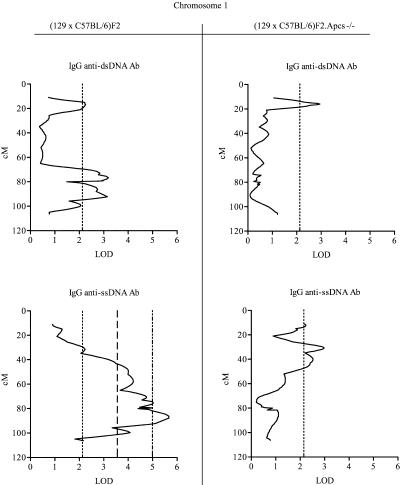
Interval Mapping Scans Showing QTL on Chromosome 1 with Anti-dsDNA and Anti-ssDNA Abs Centimorgan positions were deduced by interval mapping, anchoring marker locations to data from http://www.informatics.jax.org. Dotted lines indicate the threshold over which linkage was considered suggestive, dashed lines indicate the threshold over which linkage was considered significant, and dotted/dashed lines indicate highly significant linkage, as defined in Materials and Methods. See Table 3 for additional details.

**Figure 5:**
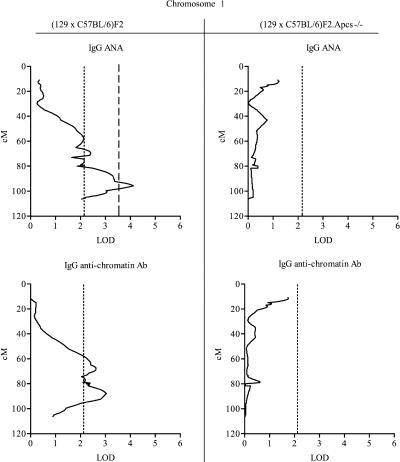
Interval Mapping Scans Showing QTL on Chromosome 1 with ANA and Anti-Chromatin Abs Centimorgan positions were deduced by interval mapping, anchoring marker locations to data from http://www.informatics.jax.org. Dotted lines indicate the threshold over which linkage was considered suggestive, and dashed lines indicate the threshold over which linkage was considered significant, as defined in Materials and Methods. See Table 3 for additional details.

The quantitative trait linkage (QTL) analysis identified several intervals on Chromosome 1 with linkage to disease serological markers, and these regions were all derived from the 129 mouse strain (see Table 3; Figures 4 and 5). Interestingly, whilst ANA and anti-chromatin Ab levels showed suggestive or significant linkages only to the telomeric region of Chromosome 1, with an estimated peak occurring at a position approximately 90 cM near the *Apcs* gene, anti-dsDNA or anti-ssDNA Ab production was also linked to other segments on Chromosome 1, indicating a more complex genetic contribution from the 129 mouse strain.

**Figure 6:**
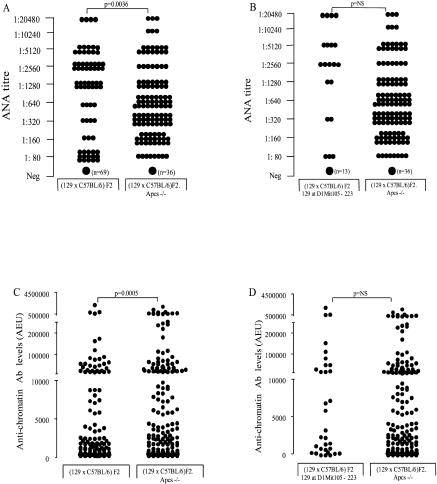
Auto-Ab Profiles (A) ANA titres in the (129 × C57BL/6)F2.*Apcs* ^−/−^ mice and (129 × C57BL/6)F2 at 1 y of age. A small circle represents one mouse; a large circle, a variable number of animals, as indicated in parentheses. Serum samples were titrated to endpoint. (B) ANA titres in the (129 × C57BL/6)F2.*Apcs* ^−/−^ mice and a selected number of wild-type (129 × C57BL/6)F2 animals carrying the Chromosome 1 region between D1Mit105 and D1Mit223 (80–106 cM) of 129 origin. The symbols are as in (A). (C and D) Anti-chromatin Ab levels expressed in AEUs related to a standard positive sample, which was assigned a value of 100 AEU. The comparison is between the same groups of mice as in (A) and (B), respectively. The symbols are as in (A).

Guided by these observations, we investigated whether the increased levels of ANA and anti-chromatin Ab observed in the *Apcs* ^−/−^ mice were caused by a gene(s) within the fixed 129 region surrounding the mutated *Apcs* gene, rather than caused by the mutated *Apcs* gene itself. We compared the levels of these auto-Abs between all (129 × C57BL/6)F2.*Apcs* ^−/−^ mice and a group of 33 wild-type mice that were selected for being homozygous 129 in the region of Chromosome 1 between microsatellites D1Mit105 and D1Mit 223 (80–106 cM) (Figure 6A–6D). In contrast to the results reported in Table 1, this comparison showed no significant differences between the two experimental groups. This result demonstrates that, most likely, the 129-derived region and not the lack of *Apcs* was mediating the production of ANA and anti-chromatin Ab. Consistent with this explanation, we found that the 129 mice have significantly higher levels of *Apcs* in circulation compared with the C57BL/6 mice (median, 83 mg/l; range, 25–208; *n* = 16 versus median, 5 mg/l; range, 4–9; *n* = 10, respectively; *p* < 0.0001). The C57BL/6 strain has previously been reported to be one of the murine strains defined as low *Apcs* producers (Pepys et al. 1979; Baltz et al. 1980). In addition, sequence analysis of the entire *Apcs* coding region in both strains failed to identify any coding sequence polymorphisms in the *Apcs* gene (data not shown), indicating that a structural variant of the protein is unlikely to be the explanation for our findings. This is consistent with a previous report by Drake et al. (1996) that showed no *Apcs* coding sequence differences amongst several autoimmune and nonautoimmune murine strains.

**Figure 7:**
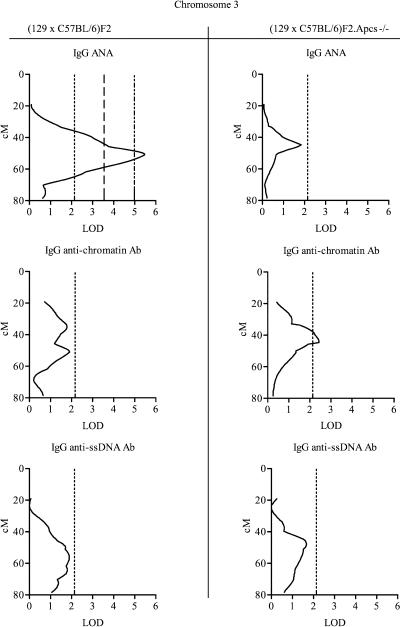
Interval Mapping Scans Showing QTL on Chromosome 3 with ANA, Anti-Chromatin, and Anti-ssDNA Abs See Table 3 for additional details. Centimorgan positions were deduced by interval mapping, anchoring marker locations to data from http://www.informatics.jax.org. Dotted lines indicate the threshold over which linkage was considered suggestive, the dashed line indicate the threshold over which linkage was considered significant, and dotted/dashed lines indicate highly significant linkage, as defined in Materials and Methods.

In addition to the 129-derived segments, in both cohorts the C57BL/6 strain contributed to the autoimmune traits with one major susceptibility locus on Chromosome 3. A genomic region between D3Mit40 and D3Mit13, with an estimated peak at position approximately 51 cM, showed a significant linkage to ANA production and weaker linkages to anti-ssDNA and anti-chromatin production (see Table 3; Figure 7). The high frequency of autoimmune phenotype in the (129 × C57BL/6) hybrid genetic background and its absence in either of the inbred parental strains imply that there are essential interactions between 129- and C57BL/6-derived alleles for the expression of autoimmunity. We investigated further the effects of genes from the C57BL/6 background by repeating the linkage analysis in (129 × C57BL/6)F2 mice, whilst controlling for the very strong 129 effect on distal Chromosome 1, as previously described (Zeng 1994). The results of this analysis showed that the statistical support for the linkage of the C57BL/6 locus on Chromosome 3 for ANA increased from logarithm of odds (LOD) 5.4 to LOD 6.4.

In contrast to these strong associations with disease serological markers, the QTL analysis identified only two potential linkages to glomerulonephritis: one in the wild-type mice on Chromosome 7 across a 10 cM region between D7Mit246 (15 cM) and D7Mit145 (26.5 cM) of 129 origin (LOD 2.86, *p* = 0.0013), and one on Chromosome 17 between D17Mit100 (11.7 cM) and D17Mit216 (29.4 cM) from the C57BL/6 strain (LOD 1.3, *p* = 0.049 and LOD 1.67, *p* = 0.021 in the wild-type and *Apcs* ^−/−^ mice, respectively). Histological evidence of glomerulonephritis was only found in approximately 20% of the mice in each cohort, which reduces the power of the QTL analysis for this disease trait.

### Production of a C57BL/6.129 Chromosome 1 Congenic Line and Its Phenotypic Analysis

We generated a C57BL/6 congenic line carrying the telomeric region of Chromosome 1 from the 129 mouse strain, in order to dissect the complex polygenic disease phenotype of the (C57BL/6 × 129/Sv)F2 hybrid strain into its individual genetic components. The 129 interval was backcrossed seven times onto C57BL/6, and at each generation the presence or absence of the Chromosome 1 interval was determined with several microsatellite markers. Each backcrossed generation was screened with more than three markers per chromosome to facilitate the removal of unselected 129 genomic regions. At the end of the backcrossing, the 129-derived Chromosome 1 interval in the congenic mice extended from microsatellite marker D1Mit105 to D1Mit223 (80–106 cM), which encompasses the most important 129 interval identified by the linkage studies in the (C57BL/6 × 129/Sv)F2 mice.

**Figure 8:**
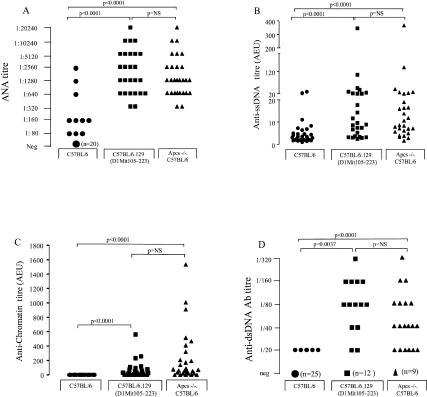
Auto-Ab Profiles (A) ANA titres in C57BL/6 mice, C57BL/6.*Apcs* ^−/−^ mice, and C57BL/6.129(D1Mit105–223) congenic mice at 1 y of age. Small symbols represent one mouse; large symbols, a variable number of animals as indicated in parentheses. Serum sample were titrated to endpoint. (B and C) Anti-ssDNA (B) and anti-chromatin (C) Ab levels in the same cohorts of mice as in (A). The Ab levels are expressed in AEUs related to a standard positive sample, which was assigned a value of 100 AEU. (D) Anti-dsDNA Ab levels. Serum samples were screened at 1:20. Samples that were positive were titrated to endpoint. The symbols are as in (A).

**Figure 9:**
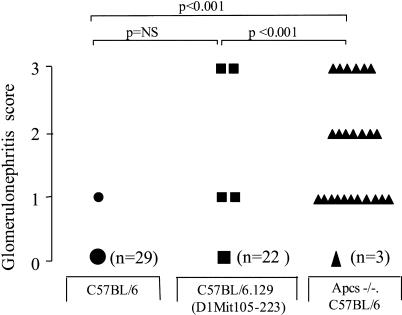
Renal Histological Assessment C57BL/6 mice, C57BL/6.*Apcs* ^−/−^ mice, and C57BL/6.129(D1Mit105–223) congenic mice were sacrificed at 1 y of age to obtain age-matched autopsy specimens. Bouin's fixed kidney sections were scored blinded for glomerulonephritis**.** Glomerulonephritis was graded on a 0–3 scale (see Materials and Methods for details).

Female Chromosome 1 congenic mice (C57BL/6.129[D1Mit105–223]), together with sex-matched *Apcs* ^−/−^ mice backcrossed onto C57BL/6 for ten generations (C57BL/6.*Apcs* ^−/−^) and C57BL/6 controls, were monitored for the presence of lupus. In the C57BL/6.*Apcs* ^−/−^ mice, the 129 genome around the *Apcs* locus was mapped as a stretch of approximately 17 cM, positioned from 87.9 cM (D1Mit15) to 105 cM (D1Mit17). Thus, the congenic line carried a similar 129 region (80–106 cM) to the one present in the C57BL/6.*Apcs* ^−/−^ mice (87.9–105 cM). At 1 y of age, all animals were sacrificed, the auto-Abs assessed, and the renal histology examined. The results of this analysis are shown in Figure 8. As previously reported (Bickerstaff et al. 1999), the levels of auto-Abs were markedly increased in the C57BL/6.*Apcs* ^−/−^ compared to the wild-type C57BL/6 controls. However, the C57BL/6.129(D1Mit105–223) animals also expressed high levels of auto-Abs, and these titres were not different from those detected in the matched congenic mice containing a null mutation of the *Apcs* gene. These results clearly demonstrated that epistatic interactions between 129 loci on Chromosome 1 and C57BL/6 genes were sufficient to mediate the loss of tolerance to nuclear autoantigens. However, in contrast to the serological data, the histological assessment of the kidneys showed evidence of markedly increased glomerulonephritis in the C57BL/6.*Apcs* ^−/−^ compared to both control groups (Figure 9), suggesting that the lack of *Apcs*, when combined with other C57BL/6 susceptibility alleles, can induce the development of severe renal damage.

## Discussion

There is accumulating evidence that background genes may influence the expression of autoimmunity in gene-targeted mice. Here we report what is to our knowledge the first systematic study that has examined this in the 129 and C57BL/6 mouse strains, widely used for gene targeting. Our results demonstrate interacting loci between 129 and C57BL/6 mice that can cause the expression of a powerful autoimmune phenotype in these animals, in the absence of any gene-targeted mutations. We also developed a congenic mouse strain bearing a portion of 129 Chromosome 1 on a C57BL/6 background and showed that this wild-type congenic line expressed striking anti-nuclear autoimmunity. By comparing this Chromosome 1 congenic strain with matched congenic mice lacking the *Apcs* gene, we demonstrated that serum amyloid P component deficiency influences the severity of glomerulonephritis, but is not the prime mover in the induction of anti-nuclear autoimmunity, contrary to our own original interpretation of our data (Bickerstaff et al. 1999). The same consideration applies to other genes located in the same Chromosome 1 region that have been implicated in the development of SLE when inactivated by gene-targeting in 129 embryonic stem cells and then backcrossed onto a pure genetic background (Bolland and Ravetch 2000; Miwa et al. 2002; Wu et al. 2002). For each, there has to be a question as to whether the anti-nuclear autoimmunity is due to the gene-targeted mutant gene or to the normal 129 genes expressed in the same region as the targeted gene.

The influence of background genes on the development of spontaneous autoimmune disease is well known, especially with respect to the *lpr* and *Yaa* disease-susceptibility genes. In MRL/Mp mice, the presence of the *lpr* gene accelerates the development of high level and broad-spectrum auto-Ab production and lethal glomerulonephritis, in addition to marked lymphoproliferative disease. In contrast, homozygosity of the *lpr* gene in other strains such as C57BL/6, AKR, LG/J, and C3H leads only to auto-Ab production (Izui et al. 1984). Similarly, the Y-chromosome-linked *Yaa* gene in BXSB and MRL/Mp males enhances the rapid development of auto-Abs and glomerulonephritis (Izui et al. 1988; Merino et al. 1989). However, in the C57BL/6 background, the *Yaa* gene does not lead to an autoimmune phenotype (Izui et al. 1988). Not surprisingly, important effects of the genetic background on the expression of autoimmunity have also been reported in gene-targeted mice (Bolland and Ravetch 2000; Santiago-Raber et al. 2001; Mitchell et al. 2002). Thus, SLE exists as a complex-trait disorder in which specific combinations of susceptibility alleles are required for the expression of the full phenotype.

Through the use of microsatellite marker maps, the identification of murine SLE susceptibility intervals in experimental crosses has been made possible. These mapping studies have shown that the disease expression in relation to the inheritance of the different alleles followed a threshold liability pattern in which a positive phenotype depended upon the presence of multiple discrete susceptibility loci with no single locus being a prerequisite factor. We have employed the same approach to analyse the genetic basis of disease inheritance in the (129 × C57BL/6) hybrid strain, the most common genetic background in gene-targeted mice. Although spontaneous autoimmunity has not been documented in either of the pure 129 or C57BL/6 strains, a spontaneous lupus-like phenotype has been described in (129 × C57BL/6) hybrid strains (Obata et al. 1979; Botto et al. 1998; Bickerstaff et al. 1999; Santiago-Raber et al. 2001), suggesting that the predisposition in these hybrid mice may arise as a result of the interaction between specific combinations of alleles inherited from both the 129 and C57BL/6 parental strains.

This was confirmed by the mapping study reported here. We showed that there are multiple genetic loci contributing to the disease and these are derived from both 129 and C57BL/6 mice. We demonstrated that a 129-derived segment of Chromosome 1 was strongly linked to the expression of auto-Abs. This region is probably capable of causing the initiation of a humoral autoimmune response to nuclear antigens; however, this response does not occur in the absence of C57BL/6 genes. In support of this, we identified a C57BL/6 segment on Chromosome 3, which may interact with the 129 genes on Chromosome 1 to mediate the loss of tolerance. Interestingly, although the C57BL/6 SLE-susceptibility region on Chromosome 3 is novel, disease-modifying alleles derived from C57BL/10 and C57BL/6 strains have been mapped to a portion of Chromosome 3 close to the region identified in this study (Morel et al. 1999; Haywood et al. 2000). Furthermore, the region on Chromosome 7 associated with the development of lupus nephritis has been linked to the same trait in other murine models of SLE (Santiago et al. 1998; Morel et al. 1999; Xie et al. 2002), suggesting the possibility of shared susceptibility loci.

Taken together our results suggest a complex genetic contribution from the (129 × C57BL/6) hybrid background genome, with both enhancing as well as inhibitory loci from the 129 mouse, in addition to genes promoting autoimmunity from the C57BL/6 mice. The impact that these interacting loci may have on the lupus-like disease present in several gene-targeted animals was further assessed by comparing *Apcs* ^−/−^ mice with Chromosome 1 genetically matched controls.

In the context of SLE susceptibility, one of the most consistently mapped non-MHC regions of the mouse genome is the telomeric Chromosome 1 segment, where several disease loci, designated *Sle1* (Morel et al. 2001), *Nba2* (Drake et al. 1995; Rozzo et al. 1996; Vyse et al. 1997), and *Bxs3* (Hogarth et al. 1998), have been mapped in lupus-prone strains. Moreover, this region of mouse Chromosome 1 is orthologous to a region on human Chromosome 1q22–1q25, which has also been linked with human SLE (Moser et al. 1998).

The *Apcs* gene is one of the candidate genes known to lie within this region. The human serum amyloid P component binds avidly to DNA, chromatin, and apoptotic cells in physiological conditions in vitro (Pepys 1974; Pepys and Butler 1987; Butler et al. 1990) and also to exposed chromatin and apoptotic cells in vivo (Hintner et al. 1988; Breathnach et al. 1989; Familian et al. 2001). We have previously reported that (129 × C57BL/6).*Apcs* ^−/−^ mice spontaneously produce a wide range of ANAs and develop significant immune complex glomerulonephritis (Bickerstaff et al. 1999). On the basis of these observations, it was postulated that *Apcs*, by altering the clearance of chromatin, contributes to the pathogenesis of SLE. However, in this study we found that only ANA and anti-chromatin Ab levels were significantly increased in the (129 × C57BL/6)F2.*Apcs* ^−/−^ mice. A possible explanation for this discrepancy may lie in the fact that the *Apcs* ^−/−^ mice analysed in the original study were generated from a limited number of founders and that this may have caused a nonrandom inheritance of the loci from the parental strains. Furthermore, the whole-genome analysis identified the 129 region surrounding the *Apcs* gene as the main locus contributing to the development of ANA and anti-chromatin Ab. In agreement with this, when we carried out a selective comparison between the (129 × C57BL/6)F2.*Apcs* ^−/−^ mice and Chromosome 1 genetically matched controls, we failed to detect any significant difference in the levels of these two auto-Abs. These findings, taken together, indicated that the phenotype associated with *Apcs* deficiency was caused by the presence of unaltered 129 genes from the telomeric region of Chromosome 1 operating in the C57BL/6 genomic background. Strong supportive evidence for this was provided by the analysis of the C57BL/6 mice congenic for this 129 region.

The generation and analysis of congenic strains have successfully been used to dissect the contribution of individual susceptibility alleles to a multigenic trait such as SLE. We adopted the same strategy to investigate the relative contribution of the 129 Chromosome 1 segment and the *Apcs* gene to each disease trait. Using this approach, we demonstrated that the 129 interval on distal Chromosome 1, when transferred onto the C57BL/6 genome, a combination commonly created by backcrossing onto C57BL/6 a mutated gene located in that chromosomal region, was sufficient to mediate the production of auto-Abs. In this context, it is of note that several strains of mice with targeted mutations of genes encoded in this region have been reported to express a lupus-like illness, including mice lacking FcγRIIB (Bolland and Ravetch 2000), complement receptors (CR1/2) (Prodeus et al. 1998; Wu et al. 2002), and decay-accelerating factor (CD55) (Miwa et al. 2002). In each case, the autoimmune phenotype was described in mice in which the null mutation was generated in 129 embryonic stem cells and then backcrossed to the C57BL/6 or another genetic background. Thus, in view of our findings, one may postulate that in each of these murine models of SLE, the effects of the targeted null gene were irrelevant. Similar conclusions may apply to other gene-targeted animals carrying mutations of genes mapped in the 129-derived susceptibility allele on Chromosome 7 (O'Keefe et al. 1996, 1999).

The expression of anti-nuclear autoimmunity was identical comparing the congenic with the *Apcs^−/−^* mice. The only difference in phenotype between these mice was in the expression of glomerulonephritis, which was more pronounced in the *Apcs^−/−^* mice compared with the congenic mice. Although these findings demonstrate that *Apcs* is not implicated in the processing of autoantigens, as it had previously been suggested, they indicate that *Apcs* might still play an important protective role in lupus nephritis. In support of this, the expression of the human C-reactive protein, an acute-phase protein closely related to *Apcs*, has been shown to delay the onset and severity of lupus nephritis in the NZB/W strain by preventing the deposition of immune complexes in the renal cortex (Szalai et al. 2003). Consistent with this, a polymorphism associated with reduced basal level of C-reactive protein has been reported to be linked to SLE in humans (Russell et al. 2004). However, as the congenic mice and the *Apcs^−/−^* mice carried similar but not identical 129 regions on Chromosome 1, an alternative explanation for our findings may still lay in the numerous and complex synergistic and counteractive interactions between 129 and C57BL/6 genes involved in self-tolerance and end organ damage. Thus, whilst the lack of lupus nephritis in the congenic mice is consistent with the need for multiple susceptibility genes for the full expression of lupus, further studies will be required to fully elucidate the role of *Apc*s in the pathogenesis of renal damage.

In summary, our findings demonstrate the impact of epistatic interactions between 129 and C57BL/6 genomes on the development of SLE and illustrate how these background gene effects may lead to incorrect interpretations when analysing the autoimmune phenotype of specific genetic disruptions.

## Materials and Methods

### 

#### Mice.

All the mice were females. Wild-type C57BL/6 and 129/Sv (129S6, according to the revised nomenclature) were bred and maintained in the animal care facility at Imperial College, London, United Kingdom. (129 × C57BL/6)F1 mice were generated by intercrossing the two wild-type strains and (129 × C57BL/6)F2 mice by interbreeding the (129 × C57BL/6)F1 mice. The *Apcs* ^−/−^mice were generated as previously reported (Botto et al. 1997), and the (129 × C57BL/6)F2.*Apcs* ^−/−^ mice were generated by intercrossing *Apcs* ^−/−^ mice on the 129 genetic background with *Apcs* ^−/−^ animals backcrossed onto C57BL/6 for ten generations. A total of 141 (129 × C57BL/6)F2 and 158 (129 × C57BL/6)F2.*Apcs* ^−/−^ female mice were produced and monitored for 1 y. Wild-type congenic C57BL/6.129(D1Mit105–223) mice were generated by backcrossing the 129 interval between microsatellites D1Mit105 and D1Mit223 (80 cM to 106 cM) onto the C57BL/6 strain. Inherited 129 regions were mapped with microsatellite markers polymorphic between 129 and C57BL/6 mice (see below). After seven generations of backcrossing, siblings were intercrossed to generate C57BL/6.129(D1Mit105–223) congenic mice homozygous for the 129 Chromosome 1 interval. Inside 129 markers at positions 81.6 cM (D1Mit159) and 105 cM (D1Mit17), respectively, and an outside C57BL/6 marker at position 74.3cM (D1Mit159) were used to further define the interval. In the C57BL/6.*Apcs* ^−/−^ mice (backcrossed onto C57BL/6 for ten generations), the 129 genome around the *Apcs* locus was mapped as a segment from 87.9 cM (D1Mit15) to 105 cM (D1Mit17). In this analysis, the inside 129 markers were at positions 93 cM (D1Mit36) and 99.7 cM (D1Mit115) and the outside C57BL/6 markers at positions 81.6 cM (D1Mit159) and 106 cM (D1Mit223). Along with 28 C57BL/6.*Apcs* ^−/−^mice and 30 C57BL/6 wild-type animals, 26 C57BL/6.129(D1Mit105–223) female mice ^−/−^were followed up to 1 y of age. Animals were maintained in specific pathogen-free conditions. All animal procedures were in accordance with institutional guidelines.

#### Serological analyses.

Sera, collected at 6 and 12 mo of age, were assayed for the presence of auto-Abs. Levels of IgG ANA were sought by indirect immunofluorescence using Hep-2 cells, and anti-dsDNA Abs were detected by indirect immunofluorescence on Crithidia luciliae as previously described (Mitchell et al. 2002). Serum samples were screened at a 1:80 (ANA) or 1:20 (anti-dsDNA) dilution and the positive samples titrated to endpoint. Abs to ssDNA and anti-chromatin were measured by ELISA, as previously described (Mitchell et al. 2002). Samples were screened at a 1:100 dilution, and the results were expressed in arbitrary ELISA units (AEUs) relative to a standard positive sample (derived from an MRL/Mp.*lpr/lpr* mouse), which was assigned a value of 100. For interplate comparison, serial dilutions of a positive control serum sample were included on each plate. Apcs levels were assessed by ELISA using sheep anti-mouse Apcs and rabbit anti-mouse Apcs Abs (Calbiochem, Nottigham, United Kingdom). Samples were screened at a 1:3,000 dilution, and the results were expressed in milligrams per liters, referring to a standard curve derived from an acute-phase serum with a known concentration of Apcs (Calbiochem). *Apcs* ^−/−^ mouse serum was included as a negative control.

#### Histological analysis.

All the mice, except the few that died before the end of the experiment, were sacrificed at 1 y of age, and kidney portions were fixed in Bouin's solution and paraffin embedded, and sections were stained with periodic acid–Schiff reagent. Glomerular histology was graded in a blinded fashion as follows: grade 0, normal; grade 1, hypercellularity involving greater than 50% of the glomerular tuft in 25%–50% of glomeruli; grade 2, hypercellularity involving greater than 50% of the glomerular tuft in 50%–75% of glomeruli; grade 3, glomerular hypercellularity in greater than 75% of glomeruli or crescents in greater than 25% of glomeruli.

#### Statistical analysis

Non-parametric data are expressed as median with range of values in parentheses. All statistics were calculated using GraphPad Prism^TM^ version 3.0 for Windows (GraphPad Software, San Diego, California, United States). Non-parametric tests were applied throughout, with differences being considered significant for *p* values < 0.05. The Mann–Whitney test was used for comparison of two groups, whilst for analysis of three groups the Kruskal–Wallis test with Dunn's multiple comparison test was used.

#### Genotypic analysis

Genotyping was carried out by PCR of genomic DNA using 143 polymorphic markers (list available on request) distributed throughout all 19 autosomes. PCRs were performed using standard reagents containing 1.5 mM MgCl_2_ and 0.4 μM primers. Microsatellite markers were screened for size polymorphisms between 129 and C57BL/6 mice. Only primers with differences detectable on ethidium bromide-stained agarose gels or on SDS-polyacrylamide gels were used.

#### Linkage analysis

The QTL program MAPMANAGER.QTL (ftp://mcbio.med.buffalo.edu/pub/MapMgr/) was used, and the two experimental groups were examined independently. Only data from mice at 12 mo of age were analysed. Log transformations of auto-Abs levels resulted in more normalised distribution and were used in QTL mapping. LOD thresholds for suggestive and significant linkages were determined by using a cohort- and trait-specific permutation test (1,000 permutations). The average threshold for suggestive, significant, and highly significant linkages were LOD ≥ 2.1 (*p* ≤ 7.8 × 10^−3^), LOD ≥ 3.6 (*p* ≤ 2.4 × 10^−4^), and LOD ≥ 5 (*p* ≤ 1 × 10^−5^), respectively (Manly and Olson 1999).

## Supporting Information

### Accession Numbers

The LocusLink (http://www.ncbi.nlm.nih.gov/LocusLink/) ID numbers for the genes and gene products discussed in this paper are *Apcs* (LocusLink ID 20219), CD35/CD21 (LocusLink ID 12902), CD55 (LocusLink ID 13136), the FAS receptor gene (LocusLink ID 14102), and FcγRII (LocusLink ID 14130).

## Abbreviations

Ab: antibody
ANA: anti-nuclear antibody
AEU: arbitrary ELISA unit
*Apcs*^−/−^: Apcs-deficient mice
*Apcs*: serum amyloid P component gene
dsDNA: double-stranded DNA
LOD: logarithm of odds
QTL: quantitative trait linkage
SLE: systemic lupus erythematosus
ssDNA: anti-single-stranded DNA
